# 17ß-Estradiol Regulates mTORC2 Sensitivity to Rapamycin in Adaptive Cardiac Remodeling

**DOI:** 10.1371/journal.pone.0123385

**Published:** 2015-04-16

**Authors:** Angelika Kusch, Maria Schmidt, Dennis Gürgen, Daniel Postpieszala, Rusan Catar, Björn Hegner, Merci M. Davidson, Shokoufeh Mahmoodzadeh, Duska Dragun

**Affiliations:** 1 Department of Nephrology and Intensive Care Medicine, Charité—Campus Virchow Klinikum, Universitätsmedizin Berlin, Berlin, Germany; 2 Center for Cardiovascular Research, Charité, Universitätsmedizin Berlin, Berlin, Germany; 3 Department of Neurology, College of Physicians and Surgeons, Columbia University, New York, New York, United States of America; 4 Max Delbrück Center for Molecular Medicine, Berlin, Germany; San Diego State University, UNITED STATES

## Abstract

Adaptive cardiac remodeling is characterized by enhanced signaling of mTORC2 downstream kinase Akt. In females, 17ß-estradiol (E2), as well as Akt contribute essentially to sex-related premenopausal cardioprotection. Pharmacologic mTOR targeting with rapamycin is increasingly used for various clinical indications, yet burdened with clinical heterogeneity in therapy responses. The drug inhibits mTORC1 and less-so mTORC2. In male rodents, rapamycin decreases maladaptive cardiac hypertrophy whereas it leads to detrimental dilative cardiomyopathy in females. We hypothesized that mTOR inhibition could interfere with 17β-estradiol (E2)-mediated sexual dimorphism and adaptive cell growth and tested responses in murine female hearts and cultured female cardiomyocytes. Under physiological in vivo conditions, rapamycin compromised mTORC2 function only in female, but not in male murine hearts. In cultured female cardiomyocytes, rapamycin impaired simultaneously IGF-1 induced activation of both mTOR signaling branches, mTORC1 and mTORC2 only in presence of E2. Use of specific estrogen receptor (ER)α- and ERβ-agonists indicated involvement of both estrogen receptors (ER) in rapamycin effects on mTORC1 and mTORC2. Classical feedback mechanisms common in tumour cells with upregulation of PI3K signaling were not involved. E2 effect on Akt-pS473 downregulation by rapamycin was independent of ERK as shown by sequential mTOR and MEK-inhibition. Furthermore, regulatory mTORC2 complex defining component rictor phosphorylation at Ser1235, known to interfere with Akt-substrate binding to mTORC2, was not altered. Functionally, rapamycin significantly reduced trophic effect of E2 on cell size. In addition, cardiomyocytes with reduced Akt-pS473 under rapamycin treatment displayed decreased SERCA2A mRNA and protein expression suggesting negative functional consequences on cardiomyocyte contractility. Rictor silencing confirmed regulation of SERCA2A expression by mTORC2 in E2-cultured female cardiomyocytes. These data highlight a novel modulatory function of E2 on rapamycin effect on mTORC2 in female cardiomyocytes and regulation of SERCA2A expression by mTORC2. Conceivably, rapamycin abrogates the premenopausal “female advantage”.

## Introduction

Mechanistic/mammalian target of rapamycin (mTOR) acts as a key regulator of cellular metabolism, growth, proliferation, survival, and differentiation in response to nutrients, energy, oxygen levels, and growth factors [1–3]. It functions as the catalytic subunit of two mTOR containing physically and functionally distinct signaling complexes—mTORC1 and mTORC2 [4]. mTORC1 consists of mTOR, raptor (regulatory associated protein of mTOR), PRAS40 (proline-rich AKT substrate 40 kDa), and mLST8 (mammalian lethal with sec-13). mTORC2 is composed of mTOR, mLST8, rictor (raptor independent companion of mTOR), mSIN1 (mammalian stress-activated protein kinase interacting protein 1), and Protor-1 (protein observed with rictor-1) and controls cell proliferation and survival by phosphorylating and activating the Akt/PKB kinase [5].

Pharmacologic inhibitors of mTOR are increasingly used in oncology and transplantation medicine, mainly due to their antiproliferative effects [6]. Rapamycin and its derivatives target primarily mTORC1 by binding to FKBP12 and either occluding or blocking the access of substrates to the active site of mTOR kinase or by disintegration of the mTORC1 complex [7]. The effect of rapamycin on mTORC2 function is far less clear and generally requires prolonged treatment in a cell type restricted manner [8]. Multiple feedback mechanisms add complexity to mTOR signaling [9]. The best characterized negative feedback loop is the inhibition of growth factor stimulation of PI3K by mTORC1 activity [10]. This feedback loop monitors and limits Akt signaling in physiological context. However, in tumor cells with high mTORC1 activation, e.g due to mutations in TSC1-TSC2-genes, the ability of insulin to stimulate PI3K/Akt signaling can be at least partially restored by prolonged rapamycin treatment [10]. Regulation of feedback loops in non-cancer cells remains unexplored. mTOR inhibitors interfere with hormonal effects as illustrated by their capability to overcome anti-estrogen resistance in breast cancer patients with hormone receptor positivity [11].

In cardiovascular medicine, drug-eluting stents with rapamycin and its derivatives are successful in the prevention of restenosis [12]. Furthermore, rapamycin has been shown to reverse maladaptive myocardial hypertrophy (MH) in hypertensive transplant patients [13]. Experimental data from different animal models focusing on maladaptive cardiovascular remodeling corroborate these findings [14–16]. The functional relevance of the specific regulation of rapamycin effects on mTORC1 and mTORC2 has not been investigated yet.

We recently described a sexual dimorphism in cardiac mTOR signaling in response to rapamycin in normotensive mice with mineralocorticoid excess induced by DOCA and salt [17]. In male DOCA mice rapamycin inhibited selectively mTORC1, upregulated mTORC2 and reversed maladaptive cardiac remodeling. In contrast, rapamycin treated female mice displayed detrimental cardiac phenotype which was attributed to strong simultaneous downregulation of both mTORC1 and mTORC2 [17]. Complete genomic deletion of mTOR resulted in a fatal, dilated-heart phenotype and even partial loss of mTOR activity impaired hypertrophic cardiac growth and accelerated heart failure development [18], implicating role of mTOR in cardiac homeostasis.

High Akt activation together with nuclear translocation has been considered as a major sex-related cardioprotective mechanism in females [19,20]. Akt is recruited to the plasma membrane by phosphatidylinositol 3-kinase (PI3K)-produced phosphatidylinositol-(3,4,5)triphosphate and then phosphorylated at T308 in the activation loop by phosphoinositide-dependent kinase 1 [21]. This induces downstream activation of the first mTOR complex mTORC1 via the tuberous sclerosis complex [22] and proline-rich Akt substrate 40kDa [23]. Complete activation of Akt kinase followed by Akt nuclear translocation and cytoprotective actions however require phosphorylation of Akt in the hydrophobic pocket at S473 by mTORC2 [5,24]. Thereby, loss of mTORC2 function in females by treatment with rapamycin could substantially contribute to maladaptive cardiac remodeling and further data are required about rapamycin related effects on mTORC2, especially in female cardiomyocytes. We addressed these issues by investigating an effect of estrogen on cellular feedback mechanisms and functional consequences induced by rapamycin in female cardiomyocytes.

## Material and Methods

### Reagents

Reagents were obtained from the following sources: Claycomb medium, phenol red-free medium 199M, and FCS from Sigma; charcoal-stripped FCS (CS-FCS) from Pan Biotech and Biochrom AG; Dulbecco´s modified Eagle´s medium/F-12 without phenol red from Life Technology; OptiMEM I from Gibco; insulin-like growth factor 1 (IGF-1) from ImmunoTools, 17β-estradiol (E2) from Sigma, diaryl-propio-nitrile (DPN) and propyl-pyrazole-trisphenol (PPT) from Tocris Bioscience; rapamycin from LC Laboratories; PD 184352 from Axonmedchem; Lipofectamine 2000 from Invitrogen; the rabbit poly- or monoclonal antibodies to mTOR (#2972), raptor (#2280), Akt-pS473 (#4060), Akt-pT308 (#2965), Akt (#9272), ERK-pT202/Y204 (#4376), ERK (#4695), GSK3αβ-pS21/9 (#9331), GSK3β (#9315), from Cell Signaling Technologies, polyconal rabbit antibody to rictor (NB100-612) from Novus Biologicus, polyclonal rabbit antibody to phospho- p70S6K-T389 (AF8963) and mouse monoclonal antibody to p70S6K (MAB8963) from R&D, polyclonal goat antibody to sarco/endoplasmic reticulum Ca^2+^-ATPase 2 (SERCA2 N19; sc-8095) from Santa Cruz, mouse monoclonal antibodies to α-actinin, DAPI, α-tubulin and ß-actin from Sigma, HRP-labeled anti-rabbit, anti-mouse and anti-goat secondary antibodies from Dianova, anti-mouse Alexa Fluor 488 and anti-rabbit Alexa Fluor 568 from Molecular Probes. Raptor siRNA and control siRNA were purchased from Santa Cruz. Poyclonal antibodies to rictor-pS1235 were a kind gift from Cell Signaling Technologies.

### In vivo rapamycin treatment

All experiments were approved by local authorities (Landesamt für Gesundheit und Soziales Berlin (Lageso); Permit Number G0028/11) and were conducted according with institutional animal care guidelines of Charité Universitätsmedizin Berlin, Germany. All efforts were made to minimize suffering. 10 week-old male and female C57Bl/6J mice were used. Rapamycin (“low concentration” 1.5 mg/kg) was administered intraperitoneally (i.p.) on every third day for up to 42 days. Serum trough levels were determined by micro-particle assay (ARCHITECT Abbott, USA) and were 4.3 ± 1.4 ng/ml.

### Preparation of cardiac tissue lysates and immunoblotting

Lysates from cardiac tissue were prepared as described previously [25]. Briefly, pieces of frozen heart tissue were transferred into tubes containing 1.4 mm ceramic lysis matrix (Precellys24, Peqlab, Erlangen, Germany) and ice cold lysis buffer (150 mM NaCl, 50 mM Tris-HCl pH 7.5, 1 mM EDTA, 1 mM EGTA, 1% Igepal CA-630, 1 mM sodium vanadate, 2 mM sodium pyrophosphate, 10 mM sodium fluoride, 10 mM ß-glycerophosphate, and complete protease inhibitor cocktail (Roche Diagnostics), 0,1% SDS). Homogenization was achieved in a tissue disruptor (FastPrep24, MPBiomedicals, USA) for 2 x 20 sec. Lysis was completed on ice for 20 min. Extracts were cleared by centrifugation and equal amounts of proteins were resolved by SDS-PAGE and transferred to PVDF membrane. Proteins were visualized by immunoblotting and enhanced chemoluminescence (ECL detection system, Thermo Scientific). Specific bands were quantified with ImageJ 1.43 software and Akt-pS473 was normalized to Akt. Data are expressed as mean ± SEM (n = 6–8 per group).

### Immunohistochemistry

Formalin-fixed and paraffin-embedded heart sections (2 μm) were dewaxed and rehydrated using a standard histology protocol. Deparaffinized sections were stained with Mayer's hemalaun (Merck, Darmstadt, Germany) for 5 s. Akt-pS473 was detected with a monoclonal rabbit antibody (clone D9E XP-TM, Cell Signaling Technology, USA) at a dilution of 1:1000. A biotinylated secondary anti-rabbit antibody and a catalyzed system (Dako, Germany) based on the streptavidin-biotin-peroxidase reaction were used for signal amplification in accordance to the instructions of the manufacturer. Isotype control IgG served as control. Stained heart sections were visualized under a light microscope (Zeiss Axio Imager A1, Carl Zeiss, Germany). For quantification of nuclear Akt-pS473 staining, a minimum of 100 nuclei per mouse heart out of randomly selected fields of view were collected for calculation of % of nuclear staining of Akt-pS473 as mean ± SEM (n = 3–5 per group).

For immunofluorescent stainings, cardiac left ventricular tissue was snap frozen in liquid nitrogen, embedded in Tissue-Tek Cryo-OCT (Fisher Scientific, Germany) compound at -10° C and cut into 8–10 μm tissue sections. After fixation in ice-cold acetone for 20 min, slides were blocked with 10% goat serum in PBS for 1 h at RT and incubated with primary antibody over night at 4°C. Fluorochrome-conjugated secondary antibody was applied for 1 h at RT. Nuclei were counterstained with DAPI. Subsequently, slides were mounted with Mowiol 4–88 mounting medium (Roth, Germany). Confocal images were acquired using a Leica TCS-SPE spectral laser scanning microscope, and images were processed by Leica Application Suite AF software (Version 1.8.0).

### Cardiomyocyte culture and treatments

The HL-1 cell line derived from murine female atrial cardiomyocytes [26] was kindly provided by W. Claycomb (Louisiana State University Health Sciences Center, New Orleans, LA, USA) on 01/21/2009. Cells were plated on gelatine/fibronectin-coated culture dishes and cultured in Claycomb medium with 10% FCS, 0.1 mM norepinephrine, 2 mM L-glutamine, 100 μg/ml penicillin/streptomycin in 5% CO_2_ at 37°C. Prior to cell stimulation with 10 nM IGF-1 for 24 h, cells were serum starved in medium 199M with 1% CS-FCS, 2 mM L-glutamine, 100 μg/ml penicillin/streptomycin (SM) for 24 h, kept serum-free (SFM) for further 24 h in medium containing additionally 10 nM E2, 10 nM PPT or 1 nM DPN as indicated. Control cells were treated with respective solvents (ethanol 10^–6^ used as solvent for E2 or 10^–6^ DMSO as solvent for PPT, DPN and rapamycin).

AC16 cells (human ventricular cardiomyocyte cell line) [27] were grown in Dulbecco´s modified Eagle´s medium/F-12 supplemented with 12.5% fetal bovine serum, penicillin/streptomycin (100units/ml/100μg/ml), and amphotericin B (0.25μg/ml) at 37°C in 5% CO_2_. For stimulation experiments, cells were serum starved in phenol red-free Dulbecco´s modified Eagle´s medium/F-12 supplemented with 2.5% charcoal stripped fetal bovine serum, penicillin/streptomycin (100units/ml/100μg/ml), and amphotericin B (0.25μg/ml) at 37°C in 5% CO_2_ for 24 h, followed by 24 h treatment with E2 (10 nM) or vehicle. Subsequently, the cells were treated with or without E2 (10 nM) and rapamycin (as indicated) for 30 min and then with IGF-1 (20 nM) for further 24 h.

For cell size determination, cells were serum starved for 24 h and then stimulated with 20 nM IGF-1 for 48 h in serum- and phenolred-free medium 199M with E2, PPT, DPN as indicated. Medium was exchanged every 24 h. Equivalent amounts of ethanol and DMSO were added to control cells. Rapamycin was used at 20 nM if not otherwise indicated and was added 30 min or indicated time prior to cell stimulations with IGF-1.

### Cell transfection

HL-1 cells were transfected with predesigned small-interfering RNA targeting rictor or negative-control siRNA using Lipofectamine 2000 in OptiMEM I. 6 h after transfection, medium was changed to SM +/- E2 for 18 h and then cells were stimulated with IGF-1 in SFM +/-E2 for 24–48 h.

### Analysis of gene expression (quantitative real-time PCR)

Total RNA was extracted with TRIzol (Invitrogen), purified and reverse transcribed into cDNA with random hexamer primers as previously described [25]. Specific oligo-nucleotide primers were synthesized by TIB MolBiol (Berlin, Germany). Primer sequences were as follows: SERCA2A (= Atp2a2, Mus musculus ATPase, Ca++ transporting, cardiac muscle, slow twitch 2, transcript variant 2, mRNA, GenBank NM_009722) sense (5´-TACTGACCCTGT CCCTGACC-3), anti-sense (5´-CTGCTCCCCAAACTCGTCTA-3). Real-time PCR was performed in the Applied Biosystems 7500 Fast Real-Time PCR system (Applied Biosystems) as described [25]. The relative amount of gene transcript was calculated by the cycle threshold method using the Applied Biosystems 7500 System v.1.2.3 software and normalized for the endogenous reference GAPDH.

### Cell lysis and immunoblotting

Cells were rinsed twice with ice-cold 10 mM HEPES, 150 mM NaCl buffer, pH 7.5 before lysis in buffer containing 40 mM TRIS/HCL, pH 8.0, 4 mM EDTA, 20% glycerol, 276 mM NaCl, 2% Triton X-100, 1 mM sodium vanadate, 2 mM sodium pyrophosphate, 10 mM sodium fluoride, 10 mM β-glycerophosphate, and complete protease inhibitor cocktail (Roche Diagnostics). After 20 min incubation on ice, the lysates were cleared by centrifugation at 14,000 rpm at 4°C for 15 min. Samples containing equal amount of proteins were resolved by SDS-PAGE and transferred to PVDF membrane. Proteins were visualized by immunoblotting and enhanced chemoluminescence (ECL detection system, Thermo Scientific). Specific bands were quantified with ImageJ 1.43 software and normalized to α-tubulin, β-actin or GAPDH as loading control and then IGF-1 induced fold stimulation calculated by ratio to value of non-stimulated control cells. Data are expressed as mean ± SEM (n = 8–14 per group).

### Cell size determination by flow cytometry

Cells were trypsinized and resuspended in PBS containing 5 mM EDTA, 2.5% FCS, and 1 μg/ml propidium iodide (PI) (Sigma). 20,000 PI-negative cells per treatment condition were analyzed in flow cytometry forward scatter (FSC-H) (FACSCalibur, Becton Dickinson). GeoMean values of histogram plots were taken for statistics. Shown are means ± SEM of at least 3 independent experiments.

### Statistical analysis

The results are expressed as means +/- SEM. Statistical analyses using two-tailed student t-test were performed with GraphPad Prism 5.0 software (GraphPad, San Diego, CA). The significance was assumed at a p value of <0.05.

## Results

### Sexual dimorphic mTORC2 activation and subcellular localization in response to rapamycin *in vivo*

**Fig 1:**
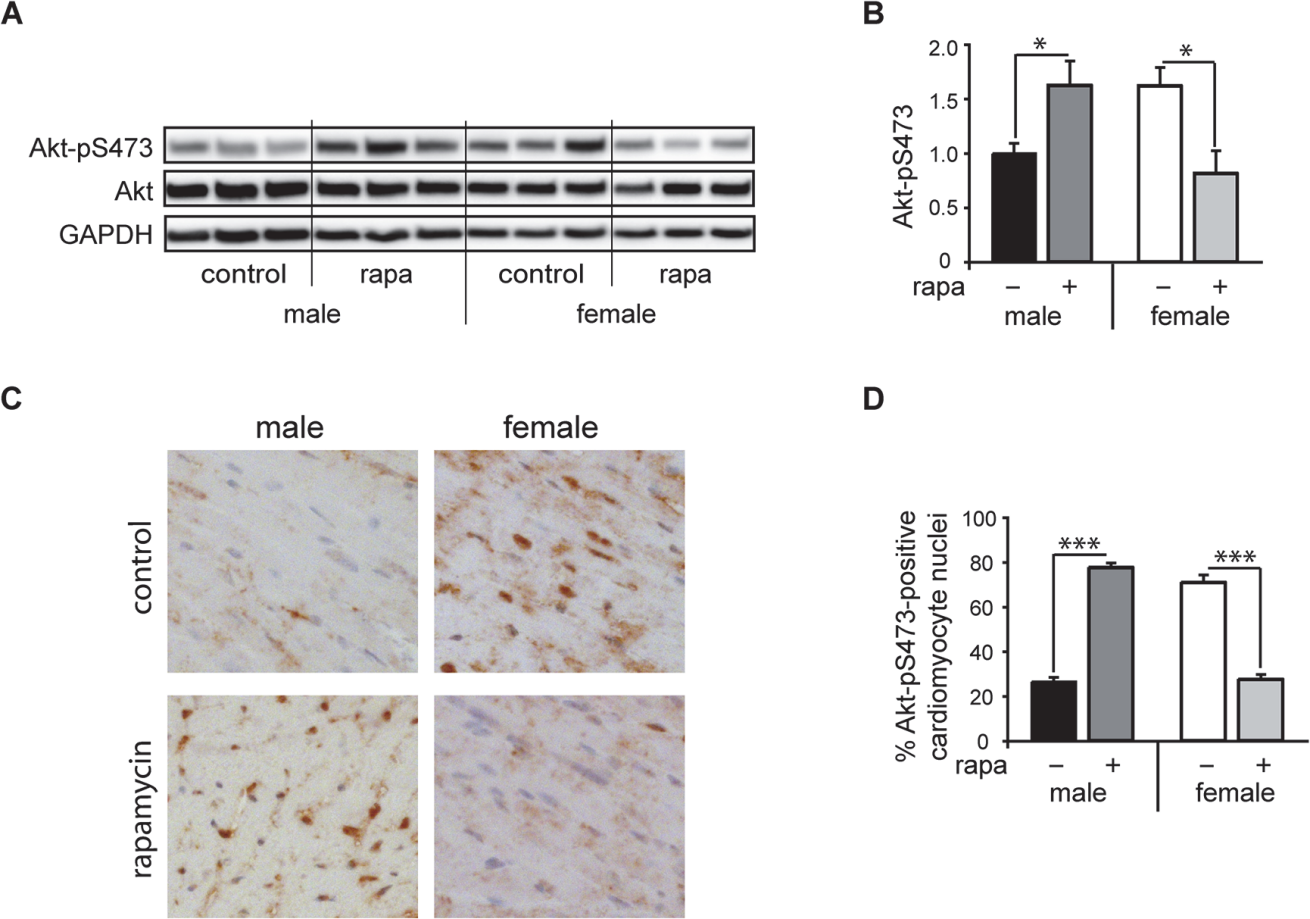
Sexual dimorphism of mTORC2 activation in response to rapamycin *in vivo*. Male and female C57Bl/6J mice were treated with rapamycin (“low concentration” 1.5 mg/kg, administered i.p. every third day) or vehicle (control) for 42 days (n = 6-8/group). mTORC2 activity was assessed by assessment of Akt phosphorylation at S473 and its nuclear localization. A, representative western blots are shown for Akt-pS473, Akt and GAPDH of mice treated as described above. B shows results from quantitative analysis of western blots for Akt-pS473 normalized to Akt (mean ± SEM; * p < 0.035) from female and male mouse hearts. C, Immunostaining for Akt-pS473 of male and female cardiac tissue sections treated with either vehicle (control) or rapamycin for 42 days and D, quantification of % cardiomyocyte nuclei stained for Akt-pS743 (mean ± SEM; *** p < 0.0001). Male mice had lower basal mTORC2 activity, yet responded to rapamycin with an increase in phosphorylation of Akt at S473, associated with increased nuclear localization important for induction of cardioprotective mechanisms. In contrast, female mice responded to rapamycin with reduced phosphorylation of Akt at S473 and loss of cardioprotective nuclear Akt.

We recently described that female mice respond to DOCA salt with higher cardiac mTORC2 activities than males and counteract this stress stimulus better than male mice. Rapamycin negatively interfered with this process by sex-specific downregulation of cardiac mTORC2 only in females [17]. To investigate, whether or not rapamycin may compromise mTORC2 function under physiological conditions, we treated male and female mice with rapamycin in the absence of pathological stimuli. mTORC2 activity was assessed by measuring Akt phosphorylation at S473 and by its intracellular localization. Male mice had lower basal activity, yet responded to rapamycin with an increase in phosphorylation of Akt at S473 (Fig 1A and 1B), associated with increased nuclear localization important for induction of cardioprotective mechanisms [28] (Fig 1C and 1D). In contrast, female mice had significant basal Akt-pS473 activity with strong nuclear localization which was progressively lost upon rapamycin treatment (Fig 1A, 1B, 1C and 1D and S1 Fig).

### Rapamycin downregulates mTORC2 in presence of E2 in female cardiomyocytes

**Fig 2:**
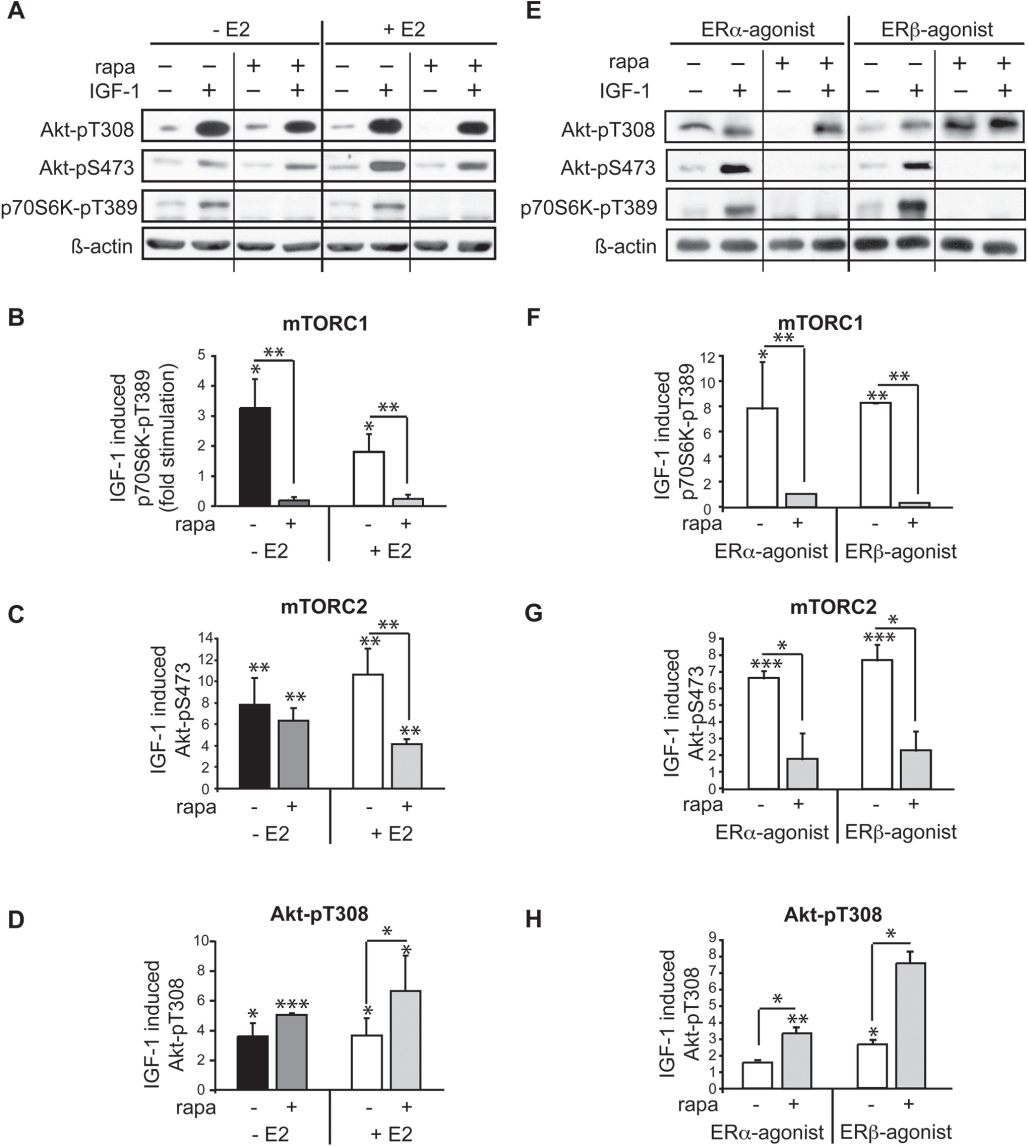
E2 regulates rapamycin effects on mTORC2 activity. A-H, Rapamycin lowers mTORC1 activity independent of presence of E2, ERα- or ERβ-agonist, however lowers mTORC2 activity dependent on presence of E2. A-D, HL-1 cells were grown to near confluence in medium containing 10 nM E2, and E-H, 10 nM ERα-agonist PPT or 1 nM ERβ-agonist DPN and serum starved for 24 hours prior to incubation with 20 nM rapamycin and IGF-1 for 24 h. A and E show representative westernblots. Equal loading was verified by blotting with antibodies against β-actin or α-tubulin. For B,C,D and F,G,H, labeled bands were quantified with ImageJ software, normalized to loading and IGF-1 induced phosphorylation of indicated proteins was determined by ratio to the value of non-IGF-1 stimulated control cells. Mean ± SEM of fold stimulation by IGF-1 is shown of at least 3 independently performed experiments. * p < 0.05,** p < 0.009, *** p < 0.0001. If not indicated differently, significances are related to respective non-IGF-1 stimulated cells.

To better understand mechanisms responsible for the sex differences in response to rapamycin, we studied the effect of the female sex hormone E2 on the regulation of feedback loops in female HL-1 cardiomyocytes under physiological conditions. For that purpose, female HL-1 cardiomyocytes were grown in the presence or absence of E2 and stimulated with IGF-1. IGF-1 significantly increased mTORC1 activation as assessed by phosphorylation of p70S6K at T389 and mTORC2 activation via Akt phosphorylation at T308 and S473 with and without E2 (Fig 2A–2D). Rapamycin completely and uniformly abolished mTORC1 activity in the presence and absence of E2. In contrast, presence of E2 was required for the rapamycin effect on mTORC2 activity. In presence of E2, rapamycin treatment lead to significantly decreased Akt-pS473 in cardiomyocytes. In the absence of E2, rapamycin had no effect on mTORC2 activity at Akt-pS473 upon IGF-1 stimulation. Interestingly, inhibition of the negative feedback loop of p70S6K on IRS1-signaling by IGF-1 appeared unaffected by E2 as demonstrated by increased canonical Akt phosphorylation at T308 upstream of mTOR in rapamycin treated cells (Fig 2A and 2D). However, this increased PI3K activity did not lead to recently reported increase in mTORC2 activation by phosphatidylinositol 3,4,5-trisphosphates [29]. These data suggest that E2 modulates rapamycin effects on mTORC2 independently from the classical negative feedback loop of mTORC1 towards mTORC2 and PI3K activity.

Cells were cultured in presence of selective ERα- (PPT) or ERβ-specific agonists (DPN), rapamycin blocked mTORC1 activity as expected and additionally Akt phosphorylation at S473 in both scenarios (Fig 2E–2G). Interestingly, rapamycin had stronger inhibitory effect on IGF-1 stimulated Akt phosphorylation at S473 in presence of both ERα- and ERß-agonists as compared to the E2 exposure. Interferences of signaling pathways induced by separate ER subtype activations or additional effects of E2 and ERα-agonist via G protein-coupled estrogen receptor-1 (GPER) may be involved [30]. Nevertheless, our results clearly point to a role of E2 via both ERα and ERß in rapamycin effects on mTORC2.

Rapamycin is known to induce release of negative feedback inhibition of mTORC1 towards IGF-1-PI3K signaling leading to enhanced Akt-pT308. Interestingly, this effect was much more pronounced in presence of ERβ-specific agonist than with ERα-specific agonist (Fig 2E and 2H). It has been described, that ERα associates with PI3K and thereby enhances further downstream signaling towards Akt-pT308. For ERβ, such association has not yet been reported, but its effect on Akt-pT308 suggests that ERβ signaling also involves PI3K activation and that specific ER act differently on mTORC1 dependent feedback loops.

HL-1 cardiomyocytes derived from atrial tissue of female hearts are broadly used cardiomyocyte cell culture model [31,32]. We extended our observation to female human ventricular cell line model of AC16 cells and obtained data consistent to the mouse cell line (S2 Fig).

### Analysis of mTOR complex protein expressions and rictor phosphorylation at Ser1235

**Fig 3:**
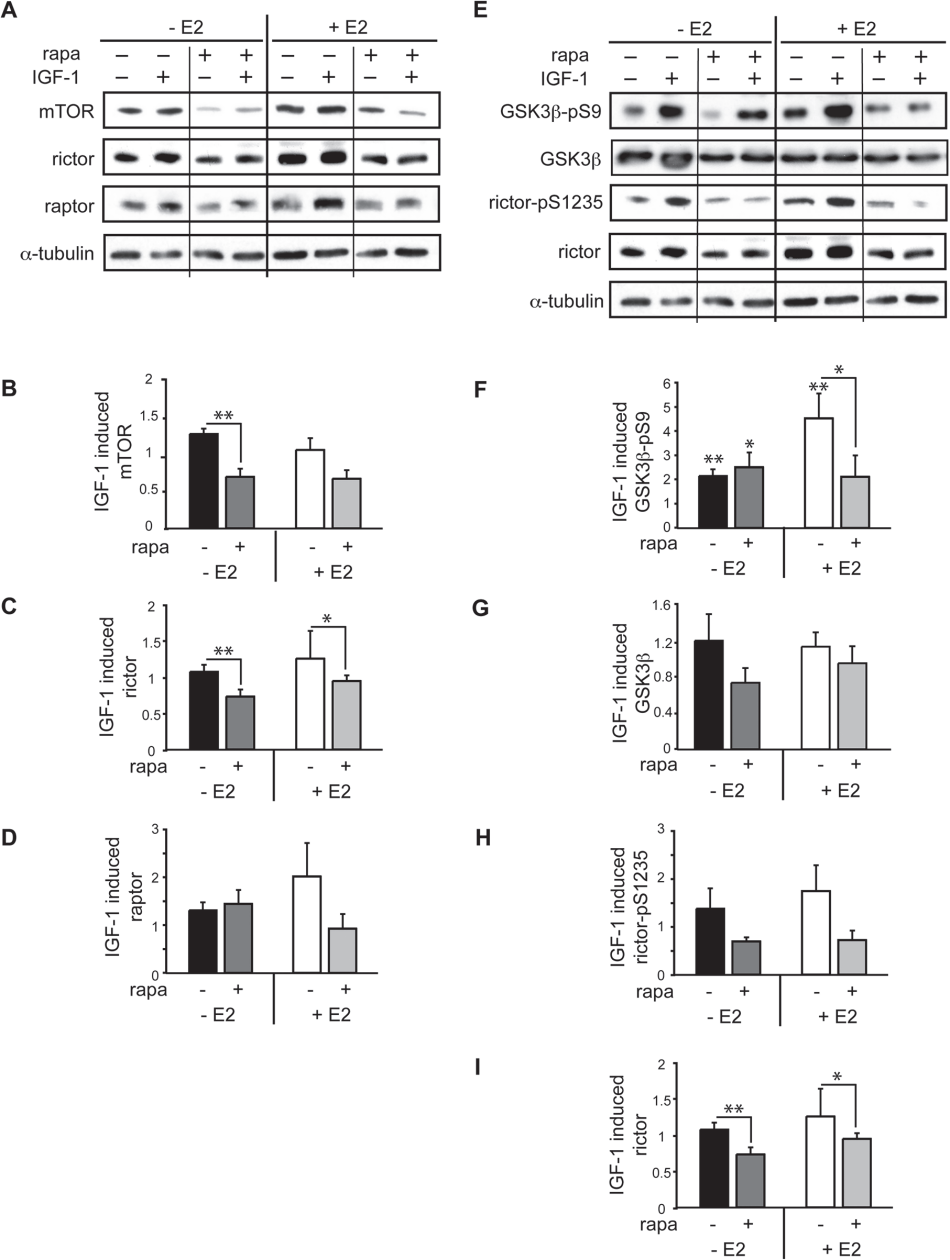
mTOR complex protein expressions and rictor phosphorylation at Ser1235. Cardiomyocytes were pretreated for 30 min with 20 nM rapamycin and then stimulated with 10 nM IGF-1 in presence or absence of 10 nM E2 for 24 h. A, shown are representative western blots for mTOR, rictor and raptor and B,C,D, quantitative analysis with mean ± SEM of fold stimulation by IGF-1 of at least 3 independently performed experiments (B,C,D). * p < 0.05, **p < 0.0095. Exposure to rapamycin lead to downregulation of mTOR and rictor, which were more pronounced under culture conditions without E2. E, Western blots for GSK3β-pS9, GSK3β, rictor pS1235 and rictor and F,G,H,I quantitative analysis of at least 3 independently performed experiments indicate that rictor phosphorylation at S1235 by GSK-3β which has been reported to interfere with Akt-substrate binding to mTORC2, thereby downregulating mTORC2 activity. IGF-1 induced strong phosphorylation of GSK-3β at Ser9 in the absence and presence of E2, however, rapamycin pretreatment only reduced this increased phosphorylation in E2 co-treated cardiomyocytes, indicating increased activity of GSK-3β. This higher activity was not associated with increased phosphorylation of rictor at S1235.

To identify regulatory mechanisms of E2 on mTORC2 sensitivity for rapamycin, we first investigated the effects of rapamycin and E2 on protein expression of mTOR, raptor and rictor. Exposure to rapamycin lead to downregulation of mTOR and rictor, which were more pronounced under culture conditions without E2 after 24 h (Fig 3A–3D). Hence, it is unlikely that differences in expression of proteins contained in mTOR complex are responsible for the attenuation of mTORC2 activity in response to rapamycin in the presence of E2. Another intriguing option for E2 modulatory action on rapamycin sensitivity of mTORC2 could be that of altered rictor phosphorylation by GSK-3β. Recent reports suggest a regulatory role of rictor phosphorylation at S1235 by GSK-3β which interferes with Akt-substrate binding to mTORC2 [33]. IGF-1 induced strong phosphorylation of GSK-3β at Ser9 in the absence and presence of E2, however, rapamycin pretreatment only reduced this increased phosphorylation in E2 co-treated cardiomyocytes, indicating increased activity of GSK-3β. Contrary to our expectations, this higher activity was not associated with increased phosphorylation of rictor at S1235, which could have explained the reduced mTORC2 activity under rapamycin plus E2 treatment (Fig 3E–3I). These results indicate that the increased GSK-3β activation is more likely a consequence of the reduced Akt activation by decreased mTORC2 activity than a regulatory role of GSK-3β in E2-mediated downregulation of mTORC2 by rapamycin. Observed increased GSK-3ß activation by rapamycin in presence of E2 is a novel finding which may essentially contribute to maladaptive cardiac remodeling as observed in female rapamycin treated mice.

### Rapamycin treatment does not influence E2 induced ERK activatio

**Fig 4:**
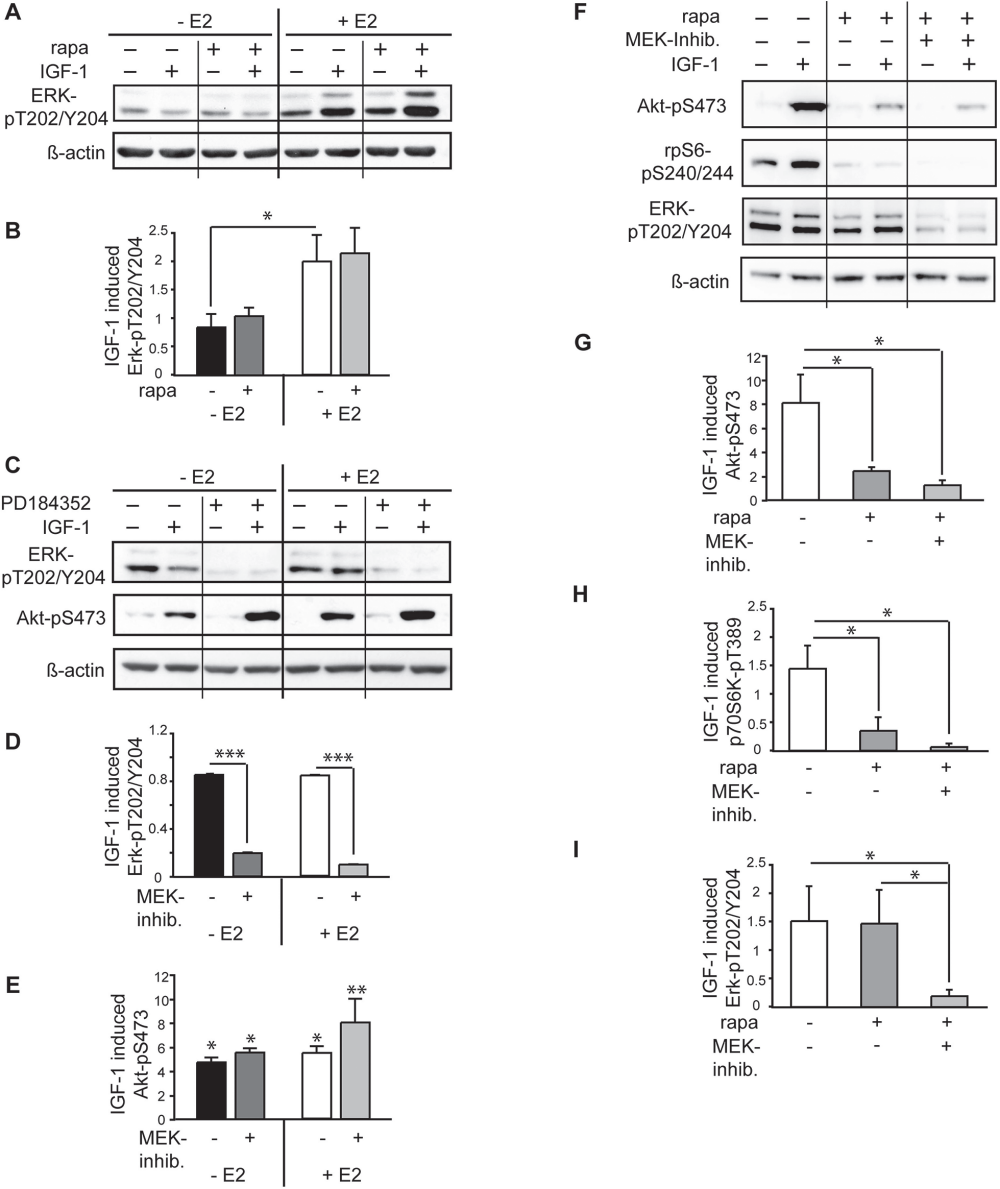
Rapamycin does not impair E2 induced ERK activation. A, ERK phosphorylation was assessed by immunoblots from lysates of HL-1 cells cultured in the presence of 10 nM E2 and treated with 20 nM rapamycin and IGF-1 for 24 h. B, Results from densitometric analysis of blots and determination of IGF-1 induced increase in protein phosphorylation levels from at least 3 independently performed experiments. C, Immunoblots and D, E, densitometric analyses of cardiomyocytes with MEK1/2 inhibition by 1μM PD 184352 1 h prior to IGF-1 stimulation resulted in increased mTORC2 activity as indicated by increased Akt-pS473 in E2 cotreated cells. * p < 0.04,** p < 0.007, *** p < 0.0007. F, Inhibition of Erk phosphorylation by MEK1/2 inhibitor PD 184352 did not inverse rapamycin effect on Akt-pS473 in E2 cultured cardiomyocytes as investigated by western blotting and G-I, Densitometric analyses; mean ± SEM of fold stimulation by IGF-1 is shown of at least 3 independently performed experiments. * p < 0.05.

MAPK signaling plays an important role in IGF-1 signaling and non-genomic E2 effects involved in physiological cell responses [34,35]. In addition, diverse cross-talks between Akt and ERK have been reported. In cancer cells, Akt inhibits the activation of the Raf-MEK-ERK pathway [36] and mTORC1 inhibition results in hyperactive RTK/IRS-1/PI3K pathway increasing the signal towards both, the mTORC2 and the Ras-Raf1-MEK1/2-ERK pathway [37]. Therefore, we evaluated the effect of E2 and rapamycin on ERK-pT202/Y204 in IGF-1 treated cardiomyocytes. E2 induced a significant increase in ERK phosphorylation (Fig 4A and 4B). Rapamycin pretreatment did not interfere with ERK activation in response to IGF-1, independent of presence of E2. However, inhibition of ERK upstream kinase MEK with pharmacologic inhibitor PD184352 in E2 cultured cardiomyocytes increased mTORC2 activity as measured by Akt-pS473 suggesting an inhibitory action of ERK on mTORC2 in presence of E2 (Fig 4C–4E). This prompted us to hypothesize that ERK participates functionally in E2 induced mTORC2 downregulation by rapamycin. However, inhibition of ERK prior to rapamycin treatment failed to reverse rapamycin induced attenuation of mTORC2 activity in E2 cultured cardiomyocytes. Thus, a pathomechanistic relevance of ERK is highly unlikely in this context (Fig 4F–4I).

### Rapamycin disturbs adaptive cellular responses of female cardiomyocytes in presence of E2

**Fig 5:**
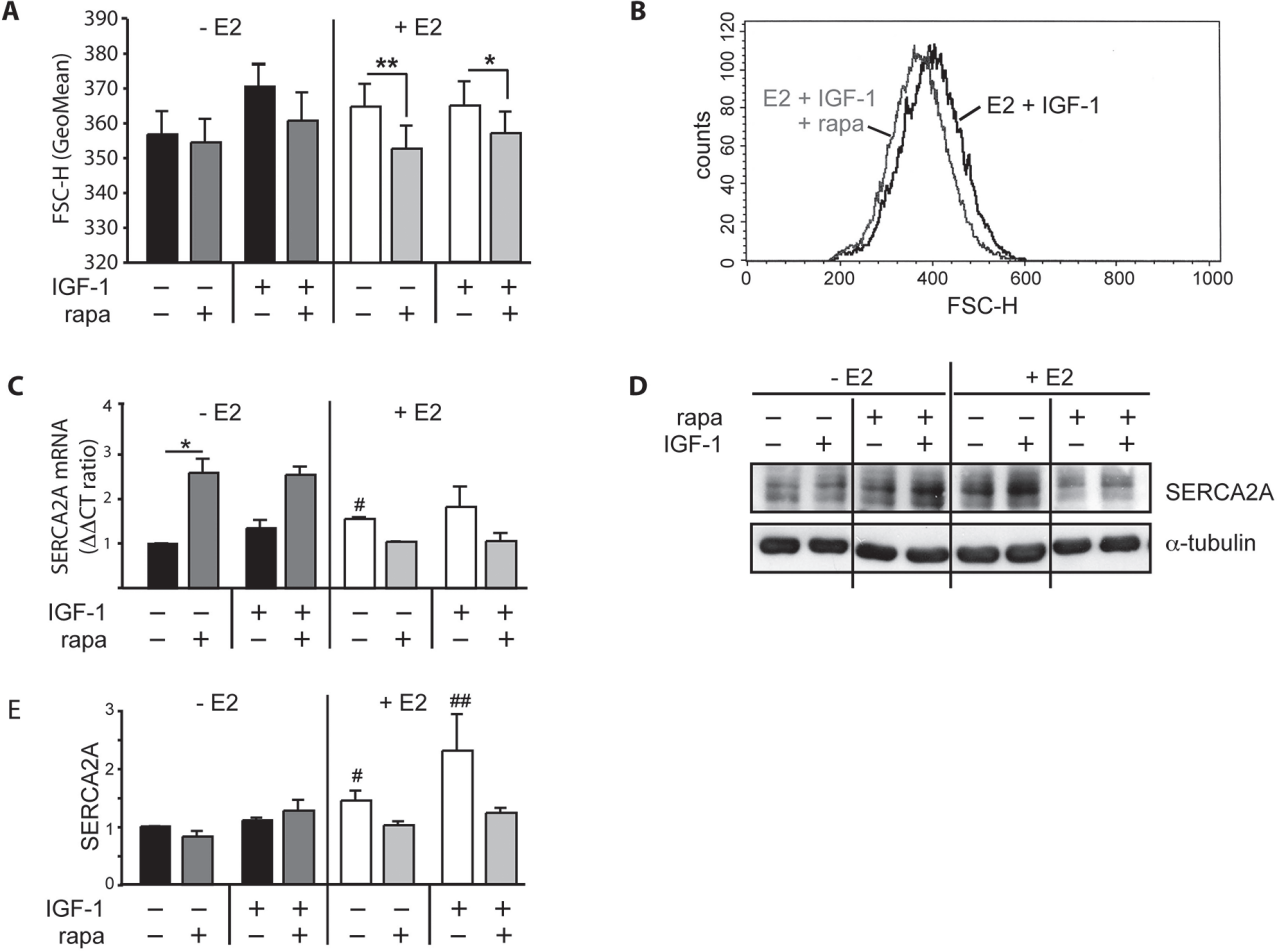
Rapamycin disturbs adaptive cardiomyocyte responses in E2 and disrupts E2-induced increase in SERCA2A expression. A, Cells underwent cell volume measurements by FACS analysis. Bars indicate mean GeoMean of flow cytometry forward scatter (FSC-H) from vital cells stimulated with 20 nM IGF-1 for 48 h as indicated from at least 3 independently performed experiments. * p < 0,00, ** p < 0,00. Rapamycin had no significant influence on cardiomyocyte cell size when E2 was absent. However, in presence of E2, rapamycin significantly decreased cardiomyocyte cell size at basal conditions and in response to IGF-1. B, representative histograms for FSC-H from cardiomyocytes cultured with 10 nM E2 and stimulated for 24 h with IGF-1 with or without prior 30 min incubation with 20 nM rapamycin showing left shift of cell volume distribution in rapamycin pretreated cells indicating decrease in cell size of all cardiomyocytes under this treatment. C, D and E, HL-1- cells were cultured with or without E2 and stimulated for 24 h with 10 nM IGF-1 with or without preincubation with 20 nM rapamycin. C-E, SERCA2A expression was assessed on C, mRNA level by qRT-PCR (values were normalized to GAPDH) and D, protein expression of cell lysates stimulated as described above. E, Densitometric analysis of immunoblots from 3 independently performed experiments shown as mean ± SEM. * p < 0.02, ** p < 0.006 for analyses as indicated; # p < 0.04, ## p < 0.006 for comparison of IGF-1 stimulated cells with E2 compared to IGF-1 stimulated cells without E2. IGF-1 alone did not significantly affect SERCA2A expression in female cardiomyocytes. However, rapamycin significantly increased SERCA2A gene expression followed by minor increases in SERCA2A protein predominantly in the IGF-1 treated cells without E2. E2 itself significantly induced SERCA2A gene expression irrespective of additional IGF-1 treatment compared to control cells without E2 (# p< 0.05). However, co-treatment with rapamycin abrogated these increases.

Rapamycin is capable to reduce cardiomyocyte hypertrophy and to increase cardiac performance in male rodents in response to maladaptive stimulus [14–16] and ameliorates pathological myocardial remodeling in cardiac transplant patients [13]. In striking contrast, rapamycin treated female mice challenged by mineralocorticoid excess develop detrimental cardiac phenotype [17]. We reasoned that rapamycin restrains adaptive cardiac responses characteristic for female sex what would lead to severe cardiac phenotype. Therefore, we assessed alterations in crucial female cardiomyocyte cell functions in response to rapamycin and E2 upon physiological stimulus. Cell size was measured in cardiomyocytes with different hormonal status subjected to 48 h IGF-1 stimulation with or without rapamycin. Rapamycin had no significant influence on cardiomyocyte cell size when E2 was absent. However, in presence of E2, rapamycin significantly decreased cardiomyocyte cell size at basal conditions and in response to IGF-1 (Fig 5A and 5B).

To gain insights into the effects of rapamycin and hormonal treatment on major proteins involved in the regulation of cardiomyocyte contractility, SERCA2A was determined on mRNA and protein level. Reduced SERCA2A levels are characteristic in human heart failure and genomic targeting aiming to increase SERCA2A expression is subject of current investigations [38,39]. IGF-1 alone did not significantly affect SERCA2A expression in female cardiomyocytes. However, rapamycin significantly increased SERCA2A gene expression followed by minor increases in SERCA2A protein predominantly in the IGF-1 treated cells without E2 (Fig 5C and 5D). E2 itself significantly induced SERCA2A gene expression irrespective of additional IGF-1 treatment compared to control cells without E2 (# p< 0.05). However, co-treatment with rapamycin abrogated these increases (Fig 5C–5E). We thus speculated that the reduced SERCA2A expression in response to rapamycin and in presence of E2 could be a consequence of reduced mTORC2 activity in cardiomyocytes.

### mTORC2 regulates SERCA2A expression in female cardiomyocytes

**Fig 6:**
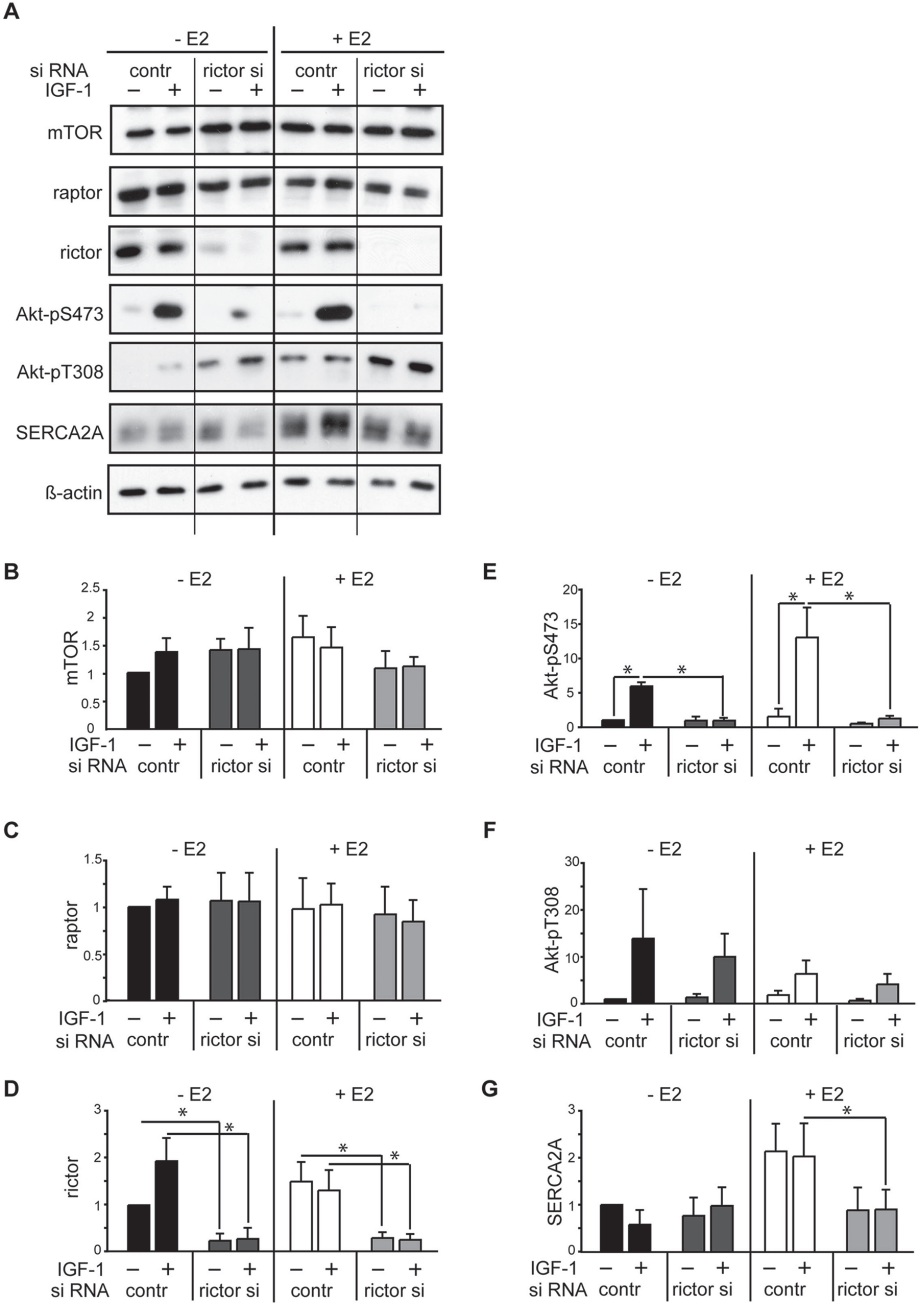
SERCA2A expression is regulated by mTORC2. Rictor silencing was induced by cell transfection with rictor siRNA and then, cells were stimulated with IGF-1 for 24 hours in presence or absence of E2. mTORC2 downregulation was confirmed by abolished Akt-pS473 and resulted in decreased SERCA2A protein expression. Akt-pT308, mTOR and raptor were not negatively affected by rictor silencing. A, Representative blots and B-G, quantitative analysis of three independently performed experiments are shown as mean ± SEM. * p < 0.043.

To prove this hypothesis, we induced rictor silencing by RNA interference in cardiomyocytes cultured in the presence and absence of E2 and stimulated them with IGF-1. As expected, IGF-1 induced strong Akt-pS473 in cells transfected with control RNA. In contrast, this phosphorylation was completely abrogated in rictor silenced cells indicating effective inhibition of mTORC2 activity (Fig 6A and 6E). Rictor silencing induced about 50% reduction of SERCA2A protein in E2 co-treated cells. These results suggest that appropriate mTORC2 function is required for E2 induced SERCA2A expression in female cardiomyocytes.

## Discussion

Our study establishes an important modulatory role of E2 in regulation of mTOR functions and cellular sensitivities towards pharmacologic mTOR inhibitors. Our results provide the first evidence that mTORC2 sensitivity towards rapamycin is modulated by E2 in cardiomyocytes and that preserved mTORC2 function is required to maintain intrinsic female cardioprotective mechanisms. Specific ERα- and ERβ-agonists completely abolished phosphorylation of Akt at S473 upon rapamycin and IGF-1 co-treatment implicating involvement of E2 via ERs.

Estrogens play a pivotal role in cardiovascular protection of premenopausal females via ER-related genomic and non-genomic actions [40]. Membrane-initiated signaling involves predominantly ERα, which functionally interacts with growth factor receptors and activates PI3K, Akt, and MAPK signaling whereas ERβ mediated nongenomic signaling is less clear [34,41]. However functionally, both ER subtypes confer protective effects in provoked cardiac pathologies [42–45]. In experimental adaptive cardiac hypertrophy, estrogens act in concert with IGF-1 to induce Akt signaling necessary for physiological growth and cell survival [19,46,47]. This cooperative action of estradiol and IGF-1 is also relevant in neuroprotection [48].

To investigate signaling mechanisms underlying these female sex-specific adaptation processes in well differentiated, non-cancerous cells, we chose HL-1 cells, which are the only immortal female cardiomyocyte cell line available that continuously divides and spontaneously contracts while maintaining phenotypic characteristics of the adult cardiomyocyte [26,31]. Although use of adult primary cardiomyocytes would at first appear more appropriate, they are not feasible for long-term experiments. Primary cardiomyocytes loose their phenotype in long-term cultures and low amount of functional cells make them extremely difficult for elaboration of cell signaling studies and genetic manipulation. Furthermore, HL-1 cardiomyocytes are increasingly well accepted for studies focusing on signal transduction [49–51]. Inclusion of human female cardiomyocytes derived from ventricular tissue (AC16 cells) confirmed our crucial results.

We carefully suggest that our data describe intrinsic E2 dependent regulation of mTOR signaling in female cardiomyocytes. Co-stimulation with E2 and IGF-1 lead to significant upregulation of mTORC2 function resulting in increased Akt phosphorylation at S473 and in addition increased ERK phosphorylation. Akt is important in physiological cardiac remodeling [46,52] and ERK activation has been mainly implicated in stress induced cardiac hypertrophy [53]. However, ERK kinase activation might also support physiological hypertrophy [54–56]. mTORC1 activity regulating protein translation in response to trophic stimuli [1] increased comparably either without hormonal co-stimulation or co-administration of ERα- or ERβ-specific agonists. In addition, E2 induced strong phosphorylation of Akt downstream kinase GSK3ß, thereby inhibiting this kinase which triggers cardioprotective mechanisms [57].

Rapamycin primarily inhibits mTORC1 and only under specific conditions or cell-type dependent mTORC2 [7,8]. Negative feedback loop of mTORC1/p70S6K towards IRS-1/PI3K signaling resulting in increased mTORC2 activity has been well described mainly in tumor cells [58,59]. Differential effects of rapamycin treatment or genomic deletions on mTORC2 downstream kinase Akt could account for the functional outcome differences seen in diverse animal models. In male mice, rapamycin effectively reduced developing or already established MH with improved cardiac function [14,16]. In contrast, genomic deletion of mTOR in the adult mouse myocardium resulted in a fatal, dilated cardiomyopathy characterized by apoptosis, autophagy, altered mitochondrial structure, and accumulation of eukaryotic translation initiation factor 4E-binding protein 1 (4E-BP1). In this model, Akt phosphorylation was remarkably increased at both, S473 and T308 [18]. Inhibition of mTORC1 in the heart by cardiomyocyte-specific deletion of raptor likewise resulted in severe dilated cardiomyopathy and in a lack of cardiac adaptive remodeling in the TAC-model in male mice [60]. Those animals similarly displayed release of negative feedback inhibition towards PI3K signaling shown by increased Akt-pT308. Concordantly, multiple previous Akt transgenic mouse models showed maladaptive cardiac remodeling, whereas temporally controlled overexpression of cardiac-specific PI3Kα seemed to be beneficial for the heart [61].

Downregulation of mTORC2 has recently been shown to result in loss of female sex-specific cardioprotection in normotensive DOCA/salt model [17]. Female mice treated with rapamycin did not only display decreased mTORC1 activity, but also reduced mTORC2 function with development of maladaptive cardiac phenotype. In contrast, male mice retained mTORC2 function upon rapamycin treatment and maintained cardiac function when challenged with mineralocorticoid [17]. Enhancement of mTORC2 function with simultaneous inhibition of mTORC1 with PRAS40 has been recently shown as protective against heart ischemic damage in male mice [32].

In physiological hypertrophy, genomic deletion of mTORC1 downstream substrate ribosomal S6 kinases alone did not attenuate physiological induced cardiac hypertrophy [62]. However, direct pharmacologic inhibition of mTORC2 downstream substrate Akt showed inhibition of physiological and aggravation of pathological hypertrophy [63]. This implicates, that a finely balanced mTORC2 activity seems to be necessary to maintain adaptive cardiac mechanisms in male mice.

We here report for the first time that rapamycin abolishes cytoprotective nuclear translocation of mTORC2 downstream kinase Akt in female mice *in vivo* and that E2 modulated sensitivities of mTORC2 for rapamycin independent from feedback loop of mTORC1 *in vitro*. Whereas IGF-1 stimulated cardiomyocytes treated with rapamycin preserved mTORC2 activity in absence of hormonal co-stimulation, cardiomyocytes co-cultured with E2 or ER-specific agonists displayed significantly decreased mTORC2 function with decreased Akt-pS472. Akt-pT308, indicative for PI3K-dependent phosphorylation remained increased under all conditions demonstrating preserved negative feedback loop of mTORC1/p70S6K towards IRS-1/PI3K signaling. Increased mTORC2 sensitivity for rapamycin upon IGF-1 stimulation occurred under treatment with both, ERα- and ERβ-specific agonists. Interestingly, Akt-pT308 increased already with ERβ-specific agonist without IGF-1 co-stimulation. Additional effects of ERβ either on regulation of feedback loop or direct PI3K signaling may be involved. Differences might also be explained by the fact that E2 and ERα-agonist PPT additionally activate GPER, whereas ERβ agonist DPN does not [30]. Rapamycin effects on Akt downstream kinase GSK3β-pS9 were surprising. We had expected maintained phosphorylation of GSK3β due to conserved Akt-pT308 in E2 cultured cardiomyocytes as phosphorylation of GSK3β has been reported to be independent from mTORC2 [64]. However, this was not the case. This rapamycin effect on GSK3β activity in presence of E2 might impair cardioprotective mechanisms in addition to those induced by mTORC2.

In contrast to mTORC1, regulation of mTORC2 activity still remains incompletely understood [9]. Positive regulation via TSC1-TSC2 complex [65,66], inhibition by Sin1 phosphorylation [67], direct activation by phosphoinositides [68] or phosphorylation of rictor at S1235 [33] have been proposed. Our data differ from increased phosphorylation of rictor at S1235 by GSK-3β as reported in HEK293T cells and tumor cell lines [33].

Our data provide evidence, that under conditions without the sex hormone E2, mTORC2 function relevant for cardiac adaptation may not be negatively influenced by rapamycin. However, presence of E2 like in premenopausal females downregulates mTORC2 for rapamycin with negative functional consequences. Female cardiomyocytes treated with rapamycin in the presence of E2 lost their capacity for adaptive growth upon IGF-stimulus and SERCA2A expression was markedly reduced. SERCA2A exerts an important homeostatic function for cardiomyocyte contractility, energetics and electrical properties and is important target in ongoing clinical studies on heart failure [38]. Growth factors and estrogens have been described to induce SERCA2A gene expression [69,70]. The effect of IGF-1 on SERCA2A protein level was minor in our cells without E2 which might be due to the fact, that HL-1 cells still have some remaining “embryonic” features, which include low SERCA2A levels. However, the effect of hormonal stimulation was significant that we consider this effect of E2 on mTORC2 and SERCA2A physiologically relevant. These differential effects of rapamycin on either increasing or decreasing SERCA2A expression dependent on the presence of E2 were surprising. Increased SERCA2A expression was observed in male mice who responded well to rapamycin *in vivo* [16] which was similar to our female cardiomyocytes which displayed maintained mTORC2 activity upon rapamycin treatment in absence of E2. Hence, we suggest that this mTORC2 dependent downregulation of SERCA2A expression is a sex-specific female mechanism mediated by estrogen effects. Long term rapamycin treatment of male aging mice with sex-steroid dysregulation due to drug-related gonadotoxic effects led to impaired cardiac function with decreased ejection fraction [71]. These findings together with our findings may implicate important interference of gonadal steroids with mTOR pathway.

## Conclusions

Our results show that in female hearts mTORC2 is differently affected by rapamycin compared to male hearts and that E2 is a crucial modulator of female cardiomyocyte response to rapamycin treatment. We are aware that we could not definitely answer why presence of E2 induced rapamycin mediated downregulation of mTORC2, yet we provide novel evidence for consequences of mTORC2 downregulation in female cardiomyocytes. Rapamycin negatively interferes with crucial cardiomyocyte functions in presence of E2 despite exposure to physiologic stimulus. Decrease in expression of proteins involved in cardiomyocyte contraction and newly described increased activation of GSK3β could ultimately lead to impaired cardiomyocyte function. In addition, we provide first evidence that E2 regulation of SERCA2A in cardiomyocytes is regulated by mTORC2, conveying a major mechanism of E2 induced adaptive responses at the level of single cardiomyocyte. Number of clinical indications for use of rapamycin derivatives is continuously increasing despite high interindividual variability in patient responses. According to our data, premenopausal females subjected to rapamycin treatment should be carefully monitored for cardiac performance.

## Supporting Information

S1 Fig E2 regulates rapamycin effects on nuclear localization of Akt in cardiomyocytes *in vivo*. Male and female C57Bl/6J mice were treated with rapamycin or vehicle control for 42 days. Cryosections were obtained from snap frozen left ventricular tissues. Cardio-myocytes were stained for α-actinin (green), Akt (red) and nuclei counterstained with DAPI (blue). Immunostainings (A) with Z-stack series (B) indicate clear nuclear localizations of Akt in cardiomyocytes predominantly in female mice under control conditions and male mice treated with rapamycin.
(TIF)

S2 Fig E2 regulates rapamycin effects on mTORC2 activity in AC16 cardiomyocytes. Rapamycin lowers mTORC1 activity independent of presence of E2, however lowers mTORC2 activity dependent on presence of E2 in a concentration dependent manner. AC16 cells were grown to near confluence in medium containing 10 nM E2 and serum starved for 24 hours prior to incubation with indicated concentrations of rapamycin and stimulation with IGF-1 (10μM) for 24 h 30 min after rapamycin administration.
(TIF)
