# Supplementary figures and images for: 17ß-Estradiol Regulates mTORC2 Sensitivity to Rapamycin in Adaptive Cardiac Remodeling
Source: PLoS One. 2015 Apr 16;10(4):e0123385. doi: 10.1371/journal.pone.0123385 (PMC4399939; doi:10.1371/journal.pone.0123385)

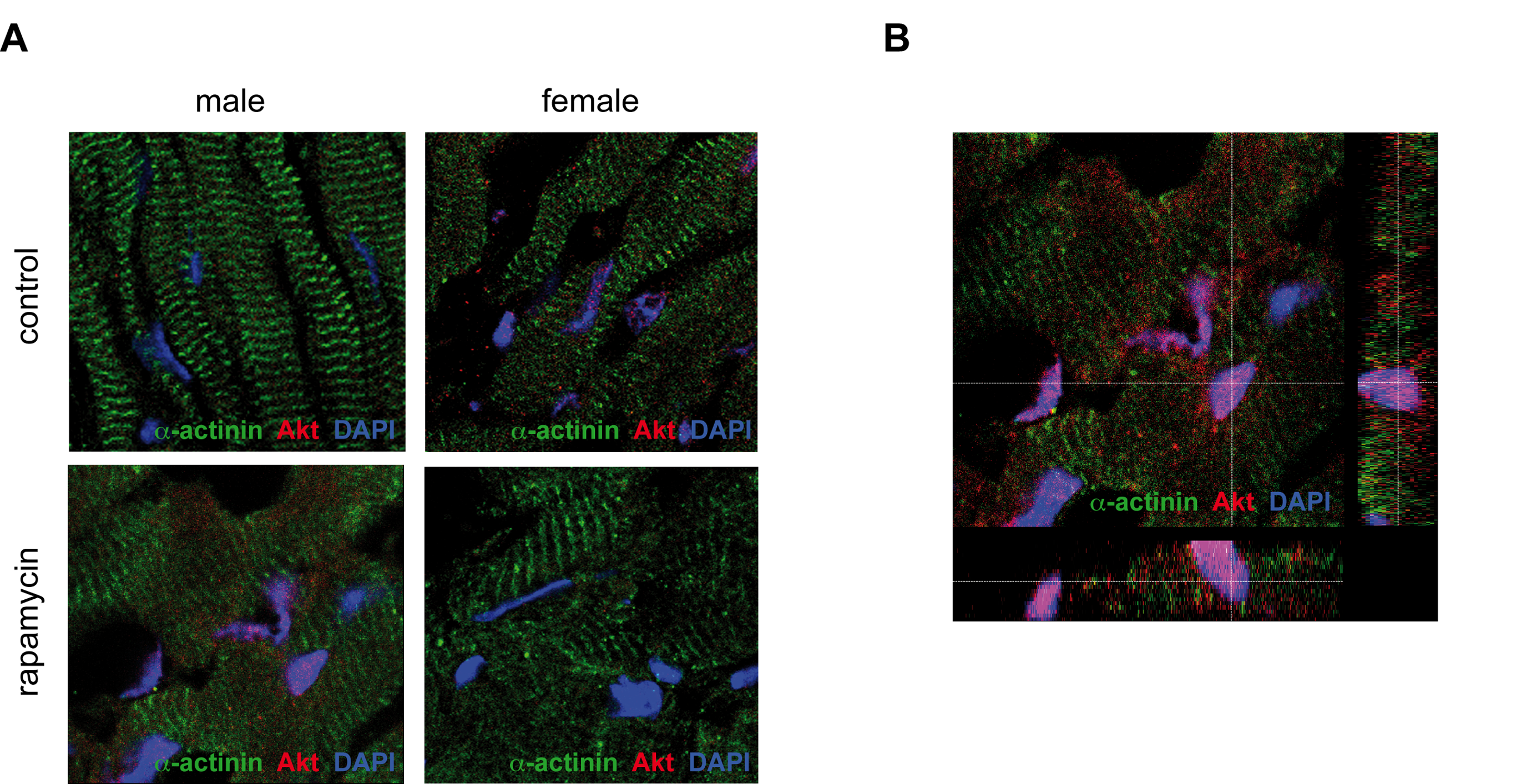

Supplement: S1 Fig — Male and female C57Bl/6J mice were treated with rapamycin or vehicle control for 42 days. Cryosections were obtained from snap frozen left ventricular tissues. Cardio-myocytes were stained for α-actinin (green), Akt (red) and nuclei counterstained with DAPI (blue). Immunostainings (A) with Z-stack series (B) indicate clear nuclear localizations of Akt in cardiomyocytes predominantly in female mice under control conditions and male mice treated with rapamycin. (TIF) [file pone.0123385.s001.tif]

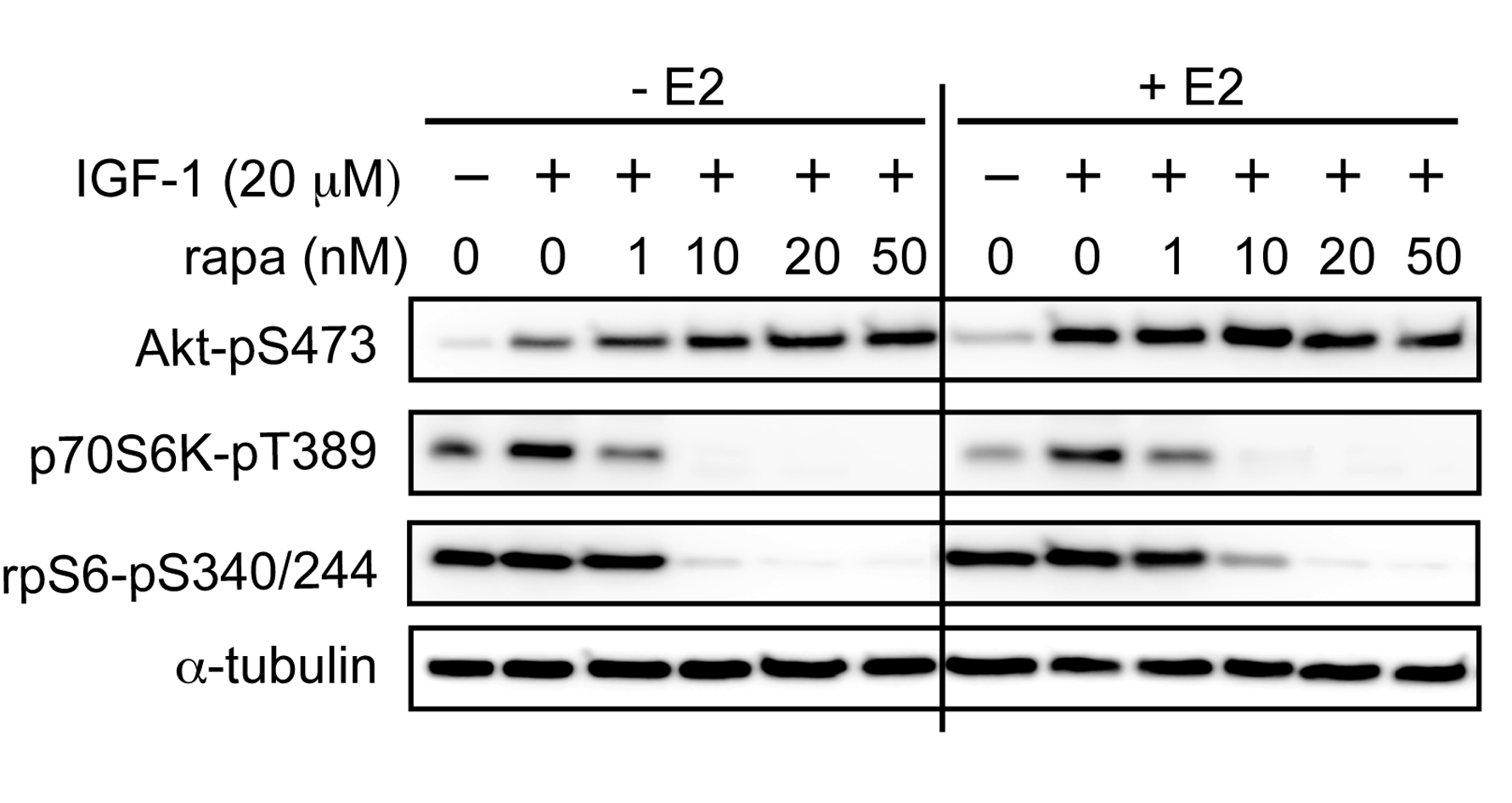

Supplement: S2 Fig — Rapamycin lowers mTORC1 activity independent of presence of E2, however lowers mTORC2 activity dependent on presence of E2 in a concentration dependent manner. AC16 cells were grown to near confluence in medium containing 10 nM E2 and serum starved for 24 hours prior to incubation with indicated concentrations of rapamycin and stimulation with IGF-1 (10μM) for 24 h 30 min after rapamycin administration. (TIF) [file pone.0123385.s002.tif]
